# 

**Published:** 1828-07

**Authors:** 


### THE LONDON MEDICAL AND PHYSICAL JOURNAL.

EDITED BY

RODERICK MACLEOD, M.D.

PHYSICIAN TO THE
WESTMINSTER GENERAL DISPENSARY,
AND LECTURER
ON THE THEORY AND PRACTICE OF PHYSIC, AND ON MATERIA MEDICA,
AT THE SCHOOL IN GREAT WINDMILL STREET:

AND

JOHN NORTH, SURGEON.

(VOL. LX.)

NEW SERIES, VOL. V.

Et quoniam variant morbi, variabimus artes;

Mille mali species, mille salutis erunt.

45593

LONDON:

JOHN SOUTER, 73, ST. PAUL'S CHURCH-YARD,

AND TO BE HAD OF ALL THE MEDICAL BOOKSELLERS.

1828.

W1
LO31

J. AND C. ADLARD, PRINTERS,
BARTHOLOMEW CLOSE.

### MONTHLY LIST OF MEDICAL BOOKS.

[*Medical Works cannot be entered on this List except a copy be sent for the purpose; the titles of Books having frequently been transmitted to us, as published, which have not appeared for weeks, or even months, after.*]

Elements of the Theory and Practice of Physic. By G. GREGORY, M.D. &c. &c. Third Edition, with numerous Additions and Amendments.—1828.

Cases of Mental Disease, with Practical Observations on the Medical Treatment. For the use of Students. By ALEXANDER MORISON, M.D. &c. &c. —8vo. pp. 164. Longman and Co. London, 1828.

Deafness, its Causes, Prevention, and Cure. By JOHN STEVENSON, Esq. —8vo. 262. Colburn, London, 1828.

On the Curative Influence of the Southern Coast of England, especially that of Hastings. With Observations on Diseases in which a Residence on the Coast is most beneficial. By WM. HARWOOD, M.D.—8vo. pp. 326. Colburn, London, 1828.

Statement by Dr. FORBES, of the Circumstances which led to his Resignation as Physician to the Royal Westminster Eye Infirmary, &c. &c.—pp. 47.

### NOTICES.

*Communications have been received from* MR. WALLACE, DR. BURNETT, *and* MR. E. S. HALL.

